# Adaptation and Validity of the Chinese Version of the Bicycle Rider Behaviour Questionnaire (BRBQ) and Its Associations with Personality Traits, Cycling Behaviours, and Traffic Crashes

**DOI:** 10.3390/bs16101775

**Published:** 2026-09-28

**Authors:** Zixu Wang, Long Sun

**Affiliations:** School of Psychology, Liaoning Normal University, Dalian 116021, China; wzx13332250929@163.com

**Keywords:** bicycle rider behaviour, personality traits, cycling behaviour, traffic crashes

## Abstract

This study conducted an initial psychometric evaluation of a translated and contextually refined Chinese-language version of the Bicycle Rider Behaviour Questionnaire (BRBQ) and examined its associations with personality traits, cycling behaviours measured by the Cycling Behaviour Questionnaire (CBQ), and self-reported traffic crashes. A total of 482 regular cyclists aged 18–60 years from four geographical regions of China were randomly divided into two subsamples (*n* = 241 each) for exploratory and confirmatory factor analyses. The analyses supported a 25-item, four-factor structure comprising traffic violations, notice failures, distracted behaviours, and control errors. Cronbach’s α coefficients ranged from 0.70 to 0.84. Average variance extracted values exceeded 0.50, and HTMT ratios were below the prespecified threshold, supporting convergent and discriminant validity. The BRBQ factors showed theoretically consistent associations with personality traits and CBQ dimensions. All four BRBQ factors were associated with retrospective crash outcomes in bivariate analyses, whereas only traffic violations remained independently associated with crash involvement after adjustment for demographic and cycling-exposure covariates. Overall, the findings provide preliminary psychometric evidence for the adapted Chinese-language BRBQ and support further independent validation.

## 1. Introduction

Bicycles remain an important mode of transport in China, and cyclist injuries continue to represent a road-safety concern (Gao et al., 2025; Li et al., 2022). Previous Chinese research has also shown that aberrant cycling behaviours are associated with self-reported crash involvement (Wang, 2019). These findings highlight the need for well-evaluated Chinese-language measures of aberrant bicycle-riding behaviour.

### 1.1. Bicycle Riding Behaviour

To assess cyclists’ on-road behaviours and elucidate their relationship with crash risk, researchers have developed several standardized measurement tools. Among these, the Cycling Behaviour Questionnaire (CBQ) and the BRBQ are the most extensively employed. Validated as a self-report instrument, the CBQ quantifies both risky and protective cycling behaviours. Its final 29-item version encompasses three factors, namely, errors, violations, and positive behaviours. Since its publication, the CBQ has been widely adopted in cycling safety research and has demonstrated robust psychometric properties across diverse cultural contexts (Useche et al., 2022). In China, CBQ-derived measures have also been employed to characterize aberrant riding patterns and their associations with self-reported traffic crashes among urban cyclists (Wang, 2019).

The BRBQ was originally developed by Hezaveh and colleagues to assess aberrant bicycle riding behaviours through the lens of human error theory. The initial instrument comprises 34 items and yields a five-factor structure: stunts and distracted behaviours, traffic violations, notice failures, control errors, and signalling violations (Hezaveh et al., 2018). Specifically, traffic violations refer to the frequency of intentionally violating traffic rules. Notice failures indicate the degree of inattention during cycling. Distracted behaviours represent the frequency of being distracted by external factors or engaging in active distractions while cycling. Control errors denote the frequency of unintentional behaviours caused by improper operation. Conceptually, the BRBQ is grounded in the classic taxonomy that distinguishes unintentional errors from deliberate violations, a framework applied widely in traffic safety research (Reason et al., 1990).

Although the CBQ offers a broad profile of both risky and positive riding behaviours, the present study required a more fine-grained assessment of aberrant riding patterns. Compared with the CBQ, the BRBQ provides a more differentiated assessment of aberrant cycling behaviours, which may help characterize behaviour-specific patterns and inform the development of targeted interventions in the Chinese context (Useche et al., 2018; Hezaveh et al., 2018). Accordingly, the BRBQ is selected for adaptation. Moreover, its factor structure aligns with the need for behaviourally specific interventions in China’s mixed-traffic environments.

### 1.2. Links Between Bicycle Riding Behaviour and Personality Traits

The evidence regarding the relationship between personality traits and bicycle riding behaviour is suggestive but not entirely consistent. In road safety research, personality is commonly viewed as a stable individual difference that shapes habitual behavioural tendencies in traffic (Antoniazzi & Klein, 2019; Yang et al., 2013). However, when the focus narrows to cycling, empirical findings diverge across studies, likely because of differences in trait operationalization, measurement tools, and contextual factors such as the traffic environment and riding modality. Despite these inconsistencies, a convergent pattern can be discerned: risk-proneness and low rule adherence are generally associated with more aberrant riding behaviours, whereas prosocial orientation tends to correlate with safer choices (Yu et al., 2023).

Moreover, sensation seeking reflects a propensity for stimulation and excitement, which may manifest in boundary-testing manoeuvres such as speeding or risky overtaking. Anger, expressed as irritability during traffic interactions, may predispose cyclists to impulsive and confrontational responses (Yang et al., 2013). In contrast, altruism describes a cooperative, other-oriented tendency that is conceptually linked to greater consideration for other road users and stronger motivation to comply with safety norms. Normlessness, or the belief that rule-breaking can be justified, is often associated with deliberate disregard for traffic rules (Yang et al., 2013). This study examined the relationships between the four personality traits and cycling behaviours to validate the use of the BRBQ among Chinese cyclists.

### 1.3. Links Between Bicycle Riding Behaviour and Traffic Crashes

Although numerous studies have demonstrated associations between cyclists’ riding behaviours and traffic crashes, the specific behavioural factors linked most strongly to traffic crashes are not entirely consistent across investigations (O’Hern et al., 2021). Overall, aberrant cycling behaviours, particularly intentional violations and certain unintentional errors, are positively associated with self-reported traffic crashes (Hezaveh et al., 2018; Wang, 2019). An original BRBQ validation study revealed that traffic violations, control errors, and notice failures were associated with cyclists’ self-reported crash outcomes, including multivehicle collisions and incidents in which the cyclist was deemed at fault (Hezaveh et al., 2018). Subsequent research has further confirmed that several BRBQ dimensions are significantly related to previous traffic crashes (Rad et al., 2024).

However, the evidence remains limited in the Chinese context. Although the CBQ has been validated across various cultural settings, this line of research is based on the CBQ rather than on the BRBQ (Useche et al., 2018; Useche et al., 2022). To our knowledge, no published study has systematically investigated whether the factor structure of the BRBQ can be replicated or culturally adapted among Chinese cyclists, a population characterized by high bicycle use, mixed-traffic conditions, heterogeneous infrastructure, and locally specific cycling norms.

### 1.4. Objectives and Hypotheses

This study had two main objectives. The first goal was to undertake an initial translation and contextual refinement of the BRBQ for Chinese cyclists and to examine its factor structure and reliability. The second goal was to evaluate multiple sources of validity evidence for the adapted Chinese BRBQ. Convergent validity was examined at the measurement-model level, discriminant validity was evaluated by examining the empirical distinctiveness of the four BRBQ factors, and nomological validity was assessed through theoretically hypothesized associations with personality traits and cycling behaviours measured by the CBQ. Criterion-related validity was evaluated through associations with retrospective traffic crash outcomes, with comparisons between cyclists with and without traffic crashes providing additional evidence of known-group validity. Two hypotheses were proposed.

**Hypothesis 1.**  *The four factors of the Chinese BRBQ (traffic violations, notice failures, distracted behaviours, and control errors) are significantly positively associated with personality traits, such as sensation seeking, anger, and normlessness, and negatively associated with altruism. Cyclists who reported traffic crashes in the past three years score significantly higher on these BRBQ factors than those without traffic crashes do (Hezaveh et al., 2018; Reason et al., 1990; Wang, 2019; Yang et al., 2013).*

**Hypothesis 2.**  *The four factors of the Chinese BRBQ are positively correlated with the risk factors for the CBQ (traffic violations and errors) and negatively correlated with CBQ positive behaviours. This prediction is grounded in the logical consistency between aberrant riding and risky behaviours, as well as the expected inverse relationship with safe behaviours, as verified in cross-cultural research (Useche et al., 2022). The hypothesized associations with personality traits and CBQ dimensions were examined as evidence of nomological validity.*

## 2. Methods

### 2.1. Participants and Procedure

Using convenience sampling, 550 participants were recruited from major cycling groups such as commuters and recreational cyclists for the study. The inclusion criteria were as follows: (1) age 18–60 years, and (2) engagement in regular cycling at least once a week in the past 3 months to ensure practical experience with cycling scenarios. Recruitment was conducted in four cities representing different geographical regions of China: Dalian in Northeast China, Xi’an in Northwest China, Zhangzhou in Southeast China, and Chengdu in Southwest China. The cities were selected to provide geographical coverage rather than to constitute a probability sample of the national cycling population.

The survey was administered via Wenjuanxing, an online questionnaire platform. Recruitment notices, which were distributed through online cycling communities, WeChat Moments, and related social network channels, described the study, stated the eligibility criteria, and provided a link to the survey. No monetary or material incentives were provided. Prior to formal data collection, an online pilot survey was conducted with 30 eligible cyclists to examine the readability of the Chinese wording, the logical flow of the questionnaire, and the approximate completion time. Feedback from the pilot survey led to revisions of ambiguous expressions and clarification of the temporal scope of key variables, including cycling frequency and crash experience. For example, the initial crash-related question, “Have you ever experienced a traffic crash while cycling?”, was revised to ask participants to report “the number of crashes experienced in the past three years,” thereby specifying both the reference period and the required response format. No items were added or deleted at this stage. The average completion time of the final questionnaire ranged from 15 to 20 min.

A total of 501 questionnaires were retrieved. Nineteen questionnaires were excluded from the data quality screening. Sixteen questionnaires were completed in less than 90 s. Because the questionnaire contained 97 scale items in addition to demographic, cycling-related, and crash-related questions, completion within 90 s allowed less than one second per scale item and was substantially shorter than the 15–20 min completion time observed in the pilot survey. Two questionnaires were excluded because core information was missing. One questionnaire was excluded because the same response option was selected for almost all items across conceptually distinct sections. Notably, repetition of the same response across ten consecutive items was not treated as sufficient evidence for exclusion on its own. After these exclusions, 482 valid questionnaires remained. Each valid case was assigned a computer-generated random number, sorted by that number, and divided into two equal subsamples (Sample 1, *n* = 241; Sample 2, *n* = 241). Sample 1 was used for the exploratory factor analysis (EFA), and Sample 2 was used for the confirmatory factor analysis (CFA).

The first sample included 128 males (53.1%) and 113 females (46.9%). With respect to traffic crashes, 118 cyclists (55 males) had not had any traffic crashes during the past three years, and 123 cyclists (73 males) had at least one traffic crash. Of these, 74 cyclists (42 males) bore primary responsibility at least once in crashes that occurred over the past three years.

The second sample included 121 males (50.2%) and 120 females (49.8%). With respect to traffic crashes, 116 cyclists (39 males) had not had any traffic crashes during the past three years, and 125 cyclists (82 males) had at least one traffic crash. Of these, 82 cyclists (42 males) bore primary responsibility at least once in crashes that occurred over the past three years (see Table 1).

Across the full sample, 248 of 482 cyclists (51.5%) reported at least one bicycle-related crash during the previous three years. The crash prevalence reported here describes the present convenience sample and should not be interpreted as a population incidence estimate. First, the crash definition was deliberately broad, covering collisions with motor vehicles, other cyclists, pedestrians, or fixed objects, and single-bicycle falls, over a three-year recall period. This scope increases the likelihood of capturing a wider range of events than more restrictive definitions. Second, the sample comprised regular cyclists (i.e., those who ride at least once per week) who were recruited through online cycling communities and social networks. Such recruitment may have attracted cyclists with greater riding exposure, stronger safety awareness, or prior traffic crashes, which could have contributed to the observed crash proportion. Importantly, because the aim of this study was to characterize the bicycle riding behaviour among active cyclists, recruiting a sample with substantial riding exposure is appropriate for validation purposes. Third, because the crash data were self-reported, the observed proportion is best interpreted as a characteristic of this sample rather than an estimate of population crash incidence.

To further verify the consistency of the distribution of demographic variables between Samples 1 and 2, chi-square tests were conducted for categorical variables (sex, educational level, and cycling frequency), and independent-sample *t* tests were performed for the continuous variables (age and years of cycling experience). No significant differences were found with respect to sex, age, educational level, cycling frequency, or years of cycling experience (sex: *χ*^2^ = 0.372, *p* = 0.542; education level: *χ*^2^ = 0.058, *p* = 0.971; cycling frequency: *χ*^2^ = 0.093, *p* = 0.954; age: *t* = −0.876, *p* = 0.381; years of cycling experience: *t* = −0.549, *p* = 0.583).

### 2.2. Measures

#### 2.2.1. BRBQ

The BRBQ was developed by Hezaveh et al. (2018) and originally contained 34 items across five factors. Responses are recorded on a five-point scale ranging from 1 (never) to 5 (nearly all of the time), with higher scores indicating more frequent engagement in the corresponding aberrant cycling behaviours.

The original BRBQ items were translated into Chinese by the research team and reviewed for semantic clarity and contextual relevance. An eight-member panel subsequently evaluated all 34 translated items in terms of cultural relevance, comprehensibility, contextual representativeness, and behavioural relevance. The panel included five traffic psychology experts with at least eight years of research experience and three frontline traffic management practitioners with at least ten years of experience in urban nonmotorized traffic management. Each item received a five-point rating and qualitative comments. The numerical ratings were used solely for descriptive purposes to support the expert review and were not treated as formal quantitative evidence of content validity. No formal item-level content validity index or interrater agreement statistic was calculated. Item-retention decisions were based jointly on the descriptive expert ratings, cultural and contextual relevance, content redundancy, and panel consensus; no single numerical cut-off was used as a mandatory deletion criterion.

Based on the panel evaluation, four items (Items 20, 25, 33, and 34) were excluded before psychometric testing, yielding a 30-item candidate pool for EFA. The excluded items and the item-specific reasons for exclusion are provided in Supplementary Table S1. The translated BRBQ wording was included in the pilot survey described in Section 2.1. The study did not employ independent parallel forward translations, formal translator reconciliation, back-translation, or cognitive interviews with members of the target population. Accordingly, the present procedure should be regarded as an initial translation and contextual refinement rather than a complete cross-cultural adaptation protocol.

#### 2.2.2. CBQ

The Chinese version of the CBQ was used to assess cyclists’ self-reported riding behaviours in daily traffic (Wang, 2019). The scale retained the original 29-item, three-factor structure, including traffic violations (8 items; e.g., “Going against the direction of traffic (wrong way)”), errors (15 items; e.g., “Unintentionally, hitting a parked vehicle”), and positive behaviours (6 items; e.g., “I avoid cycling under adverse weather conditions”). Items were rated on a 5-point scale ranging from 0 (never) to 4 (always), with higher scores indicating more frequent engagement in the corresponding behaviours. In the present study, the Cronbach’s α coefficients for traffic violations, errors, and positive behaviours were 0.947, 0.970, and 0.920, respectively.

#### 2.2.3. Personality Trait Scale

The personality scale was derived from the International Personality Item Pool (IPIP, Goldberg et al., 2006) and consists of 34 items and four factors: trait anger (10 items; e.g., “I get angry easily”), sensation seeking (10 items; e.g., “I like excitement”), altruism (10 items; e.g., “I like helping other people”), and normlessness (4 items; e.g., “You can do anything as long as you do not get into trouble”). This scale has been widely used in road safety research in China (An et al., 2023; Yang et al., 2013). Items are rated on a 5-point Likert scale ranging from 1 (strongly disagree) to 5 (strongly agree), with higher scores indicating higher levels of the corresponding personality trait. In the present study, the Cronbach’s α coefficients for trait anger, sensation seeking, altruism, and normlessness were 0.922, 0.921, 0.907, and 0.870, respectively.

#### 2.2.4. Demographic Variables

Cyclists reported demographic and cycling-related information, including sex, age, years of cycling experience, cycling frequency, weekly average cycling time and distance, typical cycling purposes, and cycling environments, which were used to characterize cycling exposure and riding context. Crash-related outcomes were assessed by asking the participants to report the number of bicycle-related traffic crashes in the past three years and the number of times they bore primary responsibility for those crashes. In this study, a traffic crash was defined as an event that occurred while cycling and involved physical contact. Examples include collisions with a motor vehicle, another bicycle/e-bike, a pedestrian, or a fixed object, as well as single-bicycle falls caused by traffic interactions or roadway/environmental conditions. Near-miss events without physical contact were not counted (Hezaveh et al., 2018; Wang, 2019).

### 2.3. Data Analysis

Data analyses were conducted using SPSS 26.0 (IBM Corp., Armonk, NY, USA) and Mplus 8.7 (Muthén & Muthén, Los Angeles, CA, USA). SPSS was used for data screening, descriptive statistics, EFA, bivariate analyses, and multivariable logistic regression. CFA was conducted in Mplus using the WLSMV estimator for ordered categorical indicators.

The 482 valid cases were randomly divided into Sample 1 (*n* = 241) for EFA and Sample 2 (*n* = 241) for CFA. The number of EFA factors was determined by jointly considering the loading pattern, simple structure, salient primary loadings, limited cross-loadings, conceptual interpretability, and consistency of item content. Item-retention decisions were then made separately on the basis of the empirical loading pattern and conceptual consistency.

Internal consistency was evaluated using Cronbach’s α and McDonald’s ω, with composite reliability (CR) reported as an additional index. Convergent validity was evaluated using average variance extracted (AVE), and discriminant validity using the heterotrait–monotrait (HTMT) ratio. Measurement invariance across sex was evaluated sequentially using configural, weak/metric, and strong/scalar models; the manuscript should state here the exact ΔCFI and ΔRMSEA decision rules and the reference that were actually used. Nomological validity was examined through correlations of the BRBQ factors with personality traits and CBQ dimensions.

Crash-related criterion evidence was examined using Spearman correlations between BRBQ factors and retrospective crash counts, Mann–Whitney U tests comparing cyclists with and without crashes, and a multivariable binary logistic regression for crash involvement (0 = no crash, 1 = at least one crash). The adjusted model included the four BRBQ factors, educational level, sex, age, weekly cycling time, and years of cycling experience. Adjusted odds ratios (aORs), 95% confidence intervals, and *p* values were reported. All 482 participants were included in these analyses.

## 3. Results

### 3.1. Item Properties

The psychometric properties of the 30 items of the BRBQ were examined using Sample 1, and the results are presented in Table 2. According to Kline (2011), items can be considered to show no substantial deviation from normality when the absolute values of skewness and kurtosis are < 3 and 7, respectively. In the present study, the skewness values of all items ranged from 0.24 to 0.73, and the kurtosis values ranged from −0.81 to 0.11. These values were well within the recommended criteria, indicating that no item exhibited extreme skewness or kurtosis. Most item-scale correlations were at or above 0.30, although several items revealed relatively weak correlations. Therefore, all 30 items were retained for the subsequent EFA and psychometric testing.

### 3.2. EFA

Using Sample 1 (*n* = 241), EFA of the 30 candidate items produced an interpretable four-factor solution. The KMO value was 0.889, and Bartlett’s test of sphericity was significant, *χ*^2^ = 3878.23, *p* < 0.001. Five items were removed after inspection of their loading patterns and conceptual consistency: Items 1, 2, and 4 had relatively weak primary loadings (0.375, 0.336, and 0.361, respectively); Item 32 showed a cross-loading pattern (0.399 on distracted behaviours and 0.327 on traffic violations); and Item 5 loaded at 0.487 on traffic violations but was inconsistent with the conceptual content of that factor. Item-specific decisions are summarized in Supplementary Table S1.

The remaining 25 items were reanalysed using the same EFA procedure. The KMO value was 0.870, and Bartlett’s test remained significant, *χ*^2^ = 2843.01, *p* < 0.001. The final four-factor solution accounted for 48.08% of the total variance. The retained item loadings are reported in Table 2.

### 3.3. CFA

CFA was conducted in Sample 2 (*n* = 241) using Mplus 8.7 and the WLSMV estimator, treating the five-point items as ordered categorical indicators. Model fit was evaluated using multiple fit indices, including χ^2^/df, comparative fit index (CFI), Tucker–Lewis index (TLI), root mean square error of approximation (RMSEA), and standardized root mean squared residual (SRMR). Model 1 specified the 30 candidate items according to the factor assignments obtained from the initial EFA. Model 2 specified the final 25 items according to the four-factor EFA solution. No correlated residuals, cross-loadings, or modification-index-driven post hoc changes were introduced in either model. Model 1 showed inadequate fit, whereas Model 2 exhibited acceptable fit (Table 3). The results regarding the measurement invariance across sex are detailed in Table 4. The path diagram is shown in Figure 1. The final 25-item Chinese and English BRBQ with factor assignment and scoring information are provided in Supplementary Table S2.

### 3.4. Reliability

The reliability analysis indicated that the Cronbach’s α coefficients of the adapted BRBQ’s four factors ranged from 0.70 to 0.84, and the corresponding McDonald’s ω coefficients ranged from 0.737 to 0.886 (see Table 2 for factor-specific values). Internal consistency was generally acceptable, although the control-errors factor showed the lowest Cronbach’s α (0.70; McDonald’s ω = 0.786). Overall, the reliability estimates were broadly comparable to those reported for the original BRBQ (Cronbach’s α = 0.76–0.84).

### 3.5. Validity

The correlations among the BRBQ, personality scale, and CBQ factors were calculated using data from Sample 2 to assess the scale’s validity. The results are presented in Table 5.

Convergent validity was evaluated using the AVE. As presented in Table 2, the AVE values for the four BRBQ factors ranged from 0.526 to 0.637. Hence, all exceeded the recommended threshold of 0.50, indicating adequate convergent validity. The CR values ranged from 0.798 to 0.892, indicating adequate CR.

Discriminant validity was assessed using the HTMT ratio. The HTMT ratios among the four BRBQ factors ranged from 0.61 to 0.74 (Table 6). Hence, all values were below the recommended threshold of 0.85 (Henseler et al., 2015), supporting the discriminant validity of the four-factor measurement model.

The four BRBQ factors were positively correlated with risky personality traits (e.g., sensation seeking) and risky CBQ factors (traffic violations and errors), and were negatively correlated with altruism and CBQ positive behaviours. The direction and overall pattern of these associations were consistent with the prespecified theoretical expectations, thereby supporting the nomological validity of the Chinese BRBQ.

### 3.6. Criterion-Related Validity: BRBQ Factors and Traffic Crashes

#### 3.6.1. Bivariate Associations with Retrospective Crash Outcomes

Spearman correlations were used to examine the associations between the four BRBQ factors, the number of crashes, and the number of cyclist-responsible crashes during the previous three years (Table 7). All four BRBQ factors were positively associated with both crash outcomes. Traffic violations showed the largest correlations with both crash measures.

#### 3.6.2. Known-Group Validity

Mann–Whitney U tests showed that cyclists reporting at least one crash had higher scores on all four BRBQ factors than cyclists reporting no crashes (Table 8). These differences provided complementary known-groups evidence for the adapted BRBQ.

#### 3.6.3. Multivariable Analysis of Crash Involvement

A multivariable binary logistic regression was conducted in the 482 participants with complete data for the outcome, the four BRBQ factors, and the covariates (Table 9). The overall model was significant, χ^2^(9) = 91.04, *p* < 0.001, with Nagelkerke R^2^ = 0.230 and a Hosmer–Lemeshow *p* value of 0.781. After adjustment for educational level, sex, age, weekly cycling time, and years of cycling experience, traffic violations remained independently associated with crash involvement (aOR = 1.690, 95% CI = 1.069–2.671, *p* = 0.025). The adjusted associations for notice failures, distracted behaviours, and control errors were not statistically significant.

## 4. Discussion

The findings provide preliminary support for a four-factor Chinese-language BRBQ, while also identifying three issues that are important for interpretation: the reduced content coverage relative to the original instrument, the comparatively weaker performance of the control-errors factor, and the difference between the bivariate and adjusted associations with retrospective crash involvement.

### 4.1. Factor Structure and Reliability

The EFA and CFA supported a 25-item, four-factor solution comprising traffic violations, notice failures, distracted behaviours, and control errors. However, this structure should not be described as a direct replication of the original five-factor BRBQ. Three signalling-related items were removed because of weak EFA loadings, and signalling violations did not remain as a separate factor. The final version therefore retains the central distinction between deliberate violations and unintentional failures or errors while providing narrower coverage of some content represented in the original instrument (Hezaveh et al., 2018).

The control-errors factor showed the weakest psychometric performance of the four subscales. Its Cronbach’s α was 0.70, and one retained item had an EFA loading of 0.37. At the same time, McDonald’s ω, composite reliability, and AVE remained within acceptable ranges. These results do not require automatic deletion of the factor, but they indicate that control-errors scores should be interpreted more cautiously and independently replicated before further item refinement is considered.

The measurement-invariance analysis across sex addresses comparability between men and women within the present Chinese sample only. It does not establish equivalence between the adapted version and the original BRBQ across languages or national groups. Cross-language score comparisons should therefore await a fuller translation/adaptation procedure and direct tests of cross-cultural measurement equivalence.

### 4.2. Convergent, Discriminant, and Nomological Validity

The convergent and discriminant validity results were consistent with the intended four-factor measurement model. The AVE values for all four BRBQ factors exceeded the recommended criterion, indicating that the items within each factor shared an adequate proportion of common variance. At the same time, the HTMT ratios remained below the recommended threshold, suggesting that traffic violations, notice failures, distracted behaviours, and control errors were empirically distinguishable despite their conceptual relatedness. Taken together, these findings provide support for treating the four factors as related but distinct components of aberrant bicycle-riding behaviour.

The associations with personality traits further supported the nomological validity of the adapted BRBQ. Higher sensation seeking, trait anger, and normlessness were generally associated with more frequent aberrant riding behaviours, whereas altruism showed negative associations with the BRBQ factors. This pattern is broadly consistent with previous evidence suggesting that risk-oriented personality characteristics are associated with less cautious or more rule-violating traffic behaviour, whereas prosocial tendencies are more closely related to safer behavioural choices (Yang et al., 2013; Yu et al., 2023). The relationships between the BRBQ and the CBQ provide additional evidence that the adapted BRBQ is located within a theoretically coherent network of cycling-behaviour constructs. All four BRBQ factors were positively associated with CBQ traffic violations and cycling errors and negatively associated with CBQ positive behaviours. This direction of association is consistent with previous research using cycling-behaviour measures across different populations and cultural settings (Useche et al., 2018, 2022). Notably, the associations with the CBQ traffic-violation and error factors were generally stronger than most associations with the personality variables. This is theoretically reasonable because the BRBQ and CBQ both assess relatively proximal cycling behaviours, whereas personality traits represent broader and more distal individual differences. Thus, the observed pattern supports the interpretation of the BRBQ as a behaviour-specific measure that is related to, but not interchangeable with, either general personality tendencies or other measures of cycling behaviour.

### 4.3. Criterion-Related Validity and Traffic Crashes

At the bivariate level, all four BRBQ factors were positively associated with retrospective crash outcomes. Traffic violations showed the strongest correlations, while the remaining three factors yielded smaller associations. The Mann–Whitney U tests provided complementary known-groups evidence, with cyclists reporting crashes scoring significantly higher on all four BRBQ factors than cyclists without crashes. In the multivariable logistic regression adjusted for demographic and cycling-related covariates, only traffic violations remained independently associated with crash involvement. Notice failures, distracted behaviours, and control errors were not significant in the adjusted model. Given the retrospective, cross-sectional design, all reported associations should be interpreted as correlational rather than predictive or causal.

The present findings establish associations between the BRBQ factors and retrospective crash outcomes but do not identify the mechanisms underlying these relationships. Although weekly average cycling time was included as an exposure-related covariate in the multivariable analysis, other potentially relevant variables, including traffic conditions, road design, riding speed, and compensatory behaviour, were not incorporated into the crash analyses. Previous studies suggest that demanding traffic environments may increase the consequences of failures in hazard monitoring and bicycle control, whereas cyclists may sometimes compensate for distraction by reducing speed or increasing visual scanning (De Waard et al., 2010; Guo et al., 2026; Pai & Jou, 2014; Sun et al., 2023; Wang, 2019; Zhang et al., 2022). Nevertheless, these mechanisms were not tested in the present study and should be treated as hypotheses derived from previous research rather than explanations supported by the current data. Future longitudinal studies should measure these contextual and behavioural variables directly and use prospective or independently verified crash records to test the proposed mechanisms and evaluate their predictive validity.

## 5. Limitations

This study has several limitations. First, participants were recruited through convenience sampling from online cycling communities and social-network channels in four Chinese cities. The sample therefore represents regular, relatively engaged cyclists rather than a probability sample of the wider Chinese cycling population. The comparatively high proportion of participants reporting a crash should be interpreted in light of this recruitment strategy, the three-year recall period, and the broad crash definition used in the study. Second, the crash measure combined collisions with motor vehicles, bicycles or e-bikes, pedestrians, and fixed objects with single-bicycle falls, but the frequency of each crash type was not recorded separately. These events may have different behavioural and contextual determinants, so future studies should record crash type, injury severity, and relevant circumstances separately.

Third, the BRBQ, CBQ, personality measures, and crash history were all obtained primarily by self-report within the same survey. Recall error, and social-desirability bias may therefore have influenced some associations. Future validation should incorporate independently verified crash records, repeated assessment, or naturalistic behavioural measures where feasible. Fourth, the cross-sectional and retrospective design does not establish temporal order or causation. The adjusted logistic regression should therefore be interpreted as evidence of an independent association with previous crash involvement rather than as a prospective prediction model. Fifth, the translation procedure did not include independent forward translations, formal reconciliation, back-translation, or cognitive interviews, and test–retest reliability was not examined. The present instrument should therefore be regarded as an initial translated and contextually refined version pending fuller linguistic validation, assessment of temporal stability, and independent cross-cultural replication.

## 6. Implications

The main implication of the present study is methodological. The 25-item, four-factor Chinese-language BRBQ provides a preliminary research measure of aberrant cycling behaviours in Chinese adult cyclists, with generally acceptable internal consistency and evidence of convergent, discriminant, and nomological validity in the present sample. The adjusted crash analysis further showed that traffic violations, among the four BRBQ factors, were independently associated with retrospective crash involvement. Accordingly, the adapted BRBQ may be useful in research examining aberrant cycling behaviours and their correlates in similar Chinese cycling populations. Because the study was cross-sectional, based largely on self-report, and used an initial translation and contextual-refinement procedure, the findings do not support causal interpretation, individual risk classification, or direct cross-language score comparison.

## 7. Conclusions

This study provides an initial psychometric evaluation of an adapted Chinese-language BRBQ in a sample of regular adult cyclists. The final 25-item instrument retained four interpretable factors and showed generally acceptable reliability and validity evidence, although the control-errors factor requires further replication. In the adjusted crash analysis, traffic violations were the only BRBQ factor independently associated with retrospective crash involvement. The adapted BRBQ should therefore be regarded as a preliminary research measure whose structure and cross-cultural equivalence require further independent validation.

## Figures and Tables

**Figure 1:**
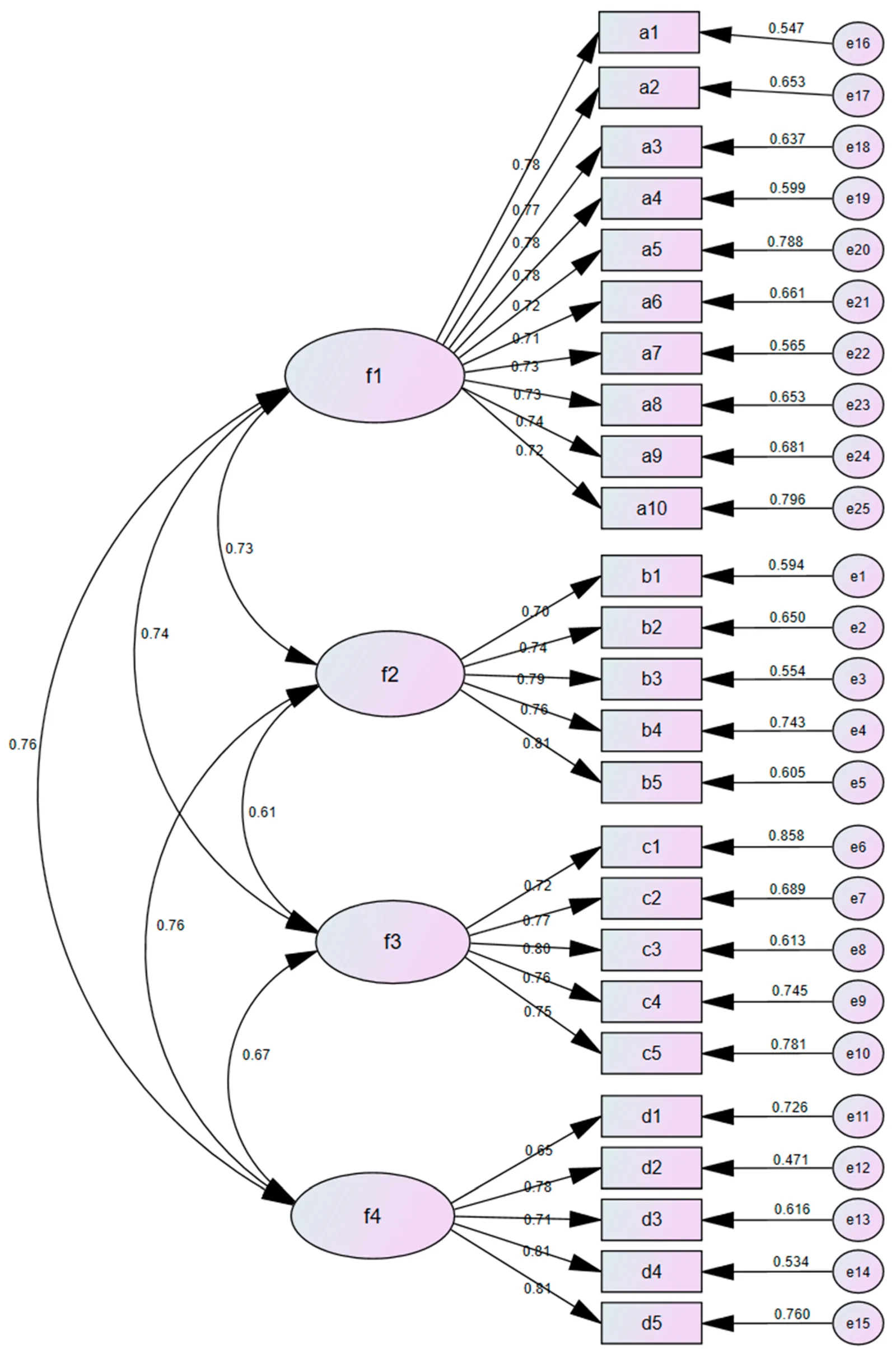
Factor loadings and covariance of the CFA (*n* = 241, f1 = traffic violations, f2 = notice failures, f3 = distracted behaviours, and f4 = control errors).

**Table 1:** Description of demographic variables.

Demographic Variable	Sample 1 (*n* = 241)				Sample 2 (*n* = 241)			
	Min	Max	Mean	SD	Min	Max	Mean	SD
Sex (female/male)	113/128				120/121			
Age (years)	18	55	29.2	9.8	18	60	30.1	10.2
Years of cycling experience (years)	1	30	6.2	5.7	2	30	6.5	5.9
Educational level	1	3	2.15	0.72	1	3	2.18	0.73
Cycling frequency	1	3	1.52	0.78	1	3	1.55	0.79
Weekly average cycling time (hours/week)	1	20	5.1	3.2	1	24	5.3	3.4

Note: educational level: 1 = junior high school, 2 = senior high school, and 3 = university and above.

**Table 2:** Item properties and EFA pattern loadings of the Chinese version of the BRBQ in Sample 1 (*n* = 241).

Item Content	Traffic Violations	Notice Failures	Distracted Behaviours	Control Errors	Communality	Mean	Standard Deviation	Item-Scale Correlation
Traffic violations (10 items, alpha = 0.84, McDonald’s ω = 0.886, AVE = 0.637, CR = 0.892)								
Speed up to pass through before the traffic light turns red	0.58				0.45	2.39	1.07	0.62 **
Run a red light	0.54				0.55	2.34	1.13	0.64 **
Ride in the opposite direction	0.57				0.61	2.40	1.13	0.75 **
Ride into restricted areas without permission from traffic signs	0.53				0.50	2.34	1.10	0.64 **
Fail to use the dedicated bicycle lane (if available)	0.49				0.35	2.27	1.07	0.52 **
Ride too close to the vehicle ahead, making it difficult to avoid in emergency situations	0.69				0.54	2.39	1.09	0.69 **
Feel angry at other road users and exhibit aggressive behaviour	0.57				0.45	2.30	1.08	0.60 **
Get angry at other road users and adopt aggressive riding styles	0.79				0.60	2.29	1.06	0.65 **
Suddenly overtake forcefully when riding at the same speed as other vehicles	0.51				0.36	2.49	1.08	0.52 **
Fail to yield to pedestrians	0.76				0.60	2.30	1.07	0.70 **
Notice failures (5 items, alpha = 0.78, McDonald’s ω = 0.794, AVE = 0.584, CR = 0.836)								
Fail to notice the vehicle ahead due to distracted behaviour or inattention		0.64			0.38	2.36	1.01	0.28 **
Fail to notice pedestrians crossing the road when turning		0.66			0.41	2.33	1.07	0.37 **
Barely have time to notice someone emerging from behind parked vehicles		0.79			0.65	2.40	1.06	0.40 **
Fail to notice pedestrians waiting to cross the road at the crosswalk		0.64			0.60	2.30	1.07	0.36 **
Barely avoid colliding with vehicles that have right of way because of failing to see the yield sign		0.54			0.46	2.31	1.08	0.37 **
Distracted behaviours (5 items, alpha = 0.75, McDonald’s ω = 0.737, AVE = 0.552, CR = 0.814)								
Listen to music while riding			0.72		0.47	2.33	1.09	0.65 **
Make phone calls while riding			0.70		0.48	2.27	0.99	0.66 **
Send text messages while riding			0.45		0.33	2.24	1.09	0.61 **
Chat with companions while riding, which distracts attention			0.54		0.41	2.31	1.10	0.66 **
Ride with at least one hand off the handlebars			0.55		0.50	2.33	1.10	0.70 **
Control errors (5 items, alpha = 0.70, McDonald’s ω = 0.786, AVE = 0.526, CR = 0.798)								
Slip on wet road surfaces or manhole covers				0.57	0.47	2.42	1.01	0.30 **
Nearly lose control due to road obstacles				0.56	0.52	2.47	1.04	0.38 **
Do not know which gear to use (for variable-speed bicycles)				0.49	0.42	2.37	1.09	0.30 **
Have difficulty maintaining balance at low speeds				0.48	0.52	2.41	1.09	0.42 **
Have difficulty controlling the speed or direction of the bicycle when going downhill				0.37	0.39	2.42	1.07	0.32 **

** *p* < 0.01. The AVE and CR were derived from the confirmed CFA model in Sample 2.

**Table 3:** Goodness-of-fit indices for the two BRBQ models in Sample 2 (*n* = 241).

Model	χ^2^	df	χ^2^/df	*p*	CFI	TLI	RMSEA	SRMR
Model 1	1193.271	405	2.946	< 0.01	0.923	0.917	0.090	0.067
Model 2	660.646	275	2.402	< 0.01	0.960	0.957	0.076	0.049

**Table 4:** Measurement invariance of the BRBQ across men and women in Sample 2 (*n* = 241).

Model	χ^2^	df	CFI	RMSEA	90% CI	SRMR	ΔCFI	ΔRMSEA
Male (*n* = 121) single group analysis	264.349	269	1.000	0.000	0.001–0.034	0.043		
Female (*n* = 120) single group analysis	333.676	269	0.960	0.045	0.026–0.060	0.055		
Configural (unconstrained)	598.025	538	0.983	0.022	0.006–0.031	0.049		
Weak measurement invariance	612.549	559	0.985	0.020	0.001–0.030	0.058	+0.0018	−0.0016
Strong measurement invariance	635.038	580	0.984	0.020	0.001–0.029	0.058	−0.0004	−0.0001

**Table 5:** Correlations among the studied variables (*n* = 241).

Variable	Traffic Violations	Notice Failures	Distracted Behaviours	Control Errors
Bicycle Rider Behaviour Questionnaire				
Traffic violations	1			
Notice failures	0.56 **	1		
Distracted behaviours	0.47 **	0.28 **	1	
Control errors	0.59 **	0.58 **	0.19 **	1
Personality traits				
Sensation seeking	0.30 **	0.31 **	0.37 **	0.34 **
Normlessness	0.19 **	0.17 *	0.28 **	0.22 **
Trait anger	0.39 **	0.34 **	0.42 **	0.39 **
Altruism	−0.29 **	−0.29 **	−0.14 **	−0.19 **
Cycling Behaviour Questionnaire				
Traffic violations	0.80 **	0.67 **	0.46 **	0.68 **
Cycling errors	0.82 **	0.69 **	0.47 **	0.70 **
Positive behaviours	−0.45 **	−0.34 **	−0.33 **	−0.49 **

* *p* < 0.05, ** *p* < 0.01.

**Table 6:** Discriminant validity–HTMT ratio.

BRBQ Factor	Traffic Violations	Notice Failures	Distracted Behaviours	Control Errors
Traffic violations	–			
Notice failures	0.72	–		
Distracted behaviours	0.74	0.74	–	
Control errors	0.74	0.61	0.67	–

**Table 7:** Spearman correlation analysis between the BRBQ factors and crash variables (*n* = 482).

Variable	Traffic Violations	Notice Failures	Distracted Behaviours	Control Errors
Number of crashes	0.24 **	0.20 **	0.16 **	0.19 **
Responsible crashes	0.24 **	0.18 **	0.17 **	0.20 **

** *p* < 0.01.

**Table 8:** Results of the Mann–Whitney U test for the BRBQ factors between groups with and without traffic crashes (*n* = 482).

BRBQ Factor	Group	Number of Cases	Mean Rank	Mann–Whitney U	Z-Value	*p*-Value	Effect Size (r)	95% CI
Traffic violations	No crash	234	208.67	21,306.50	−5.061	0.001	0.231	0.143–0.315
	With crash	248	272.74					
Notice failures	No crash	234	211.46	21,962.00	−4.646	0.001	0.212	0.125–0.297
	With crash	248	270.09					
Distracted behaviours	No crash	234	218.51	23,620.00	−3.552	0.001	0.162	0.074–0.247
	With crash	248	263.37					
Control errors	No crash	234	213.92	22,540.50	−4.264	0.001	0.194	0.106–0.280
	With crash	248	267.74					

**Table 9:** Adjusted associations between BRBQ factors and crash involvement in the multivariable logistic regression analysis (*n* = 482).

BRBQ Factor	B	SE	Wald χ^2^	aOR	95% CI	*p*
Traffic violations	0.524	0.234	5.038	1.690	1.069–2.671	0.025
Notice failures	0.251	0.191	1.728	1.285	0.884–1.868	0.189
Distracted behaviours	−0.057	0.184	0.098	0.944	0.659–1.353	0.754
Control errors	−0.045	0.183	0.061	0.956	0.668–1.368	0.805

Note. Crash involvement was coded as 0 = no crash and 1 = at least one crash during the previous three years.

## Data Availability

The original data presented in this study are openly available in Figshare at https://doi.org/10.6084/m9.figshare.32991527.

## Supplementary Materials

The following supporting information can be downloaded at https://www.mdpi.com/article/10.3390/bs16101775/s1, Table S1: Original 34-item BRBQ and adapted Chinese version with item-retention decisions; Table S2: Final 25-item Chinese and English BRBQ with factor assignment and scoring information.
