# Supplementary material for: Adaptation and Validity of the Chinese Version of the Bicycle Rider Behaviour Questionnaire (BRBQ) and Its Associations with Personality Traits, Cycling Behaviours, and Traffic Crashes
Source: Behav Sci (Basel). 2026 Sep 28;16(10):1775. doi: 10.3390/bs16101775 (PMC13701944; doi:10.3390/bs16101775)
Supplement: Supplementary file 1 [file behavsci-16-01775-s001.zip › behavsci-4470760-supplementary.pdf]

Supplementary Material

Adaptation and validity of the Chinese version of the Bicycle Rider Behaviour Questionnaire (BRBQ) and its associations with personality traits, cycling behaviours, and traffic crashes

Contents

Supplementary Table S1. Original 34-item BRBQ and adapted Chinese version with item-retention decisions

Supplementary Table S2. Final 25-item Chinese and English BRBQ with factor assignment and scoring information

Supplementary Table S1. Original 34-item BRBQ and adapted Chinese version with item-retention decisions

Original No.	Final No.	Original BRBQ item (English)	Chinese version of the BRBQ	Status	Rationale for retention/exclusion
1		Fail to give an appropriate stopping signal.	未发出适当的停车信号。	Removed	Removed after ordinal EFA because the item showed a weak primary loading (0.375). It assessed a signalling omission and did not contribute clearly to the retained four-factor structure.
2		Fail to give an audible warning when overtaking pedestrians.	超越行人时未发出可听见的警示信号。	Removed	Removed after ordinal EFA because the item showed a weak primary loading (0.336). It assessed a signalling/communication behaviour and did not contribute clearly to the retained four-factor structure.
3	16	Listen to music while riding.	骑行时听音乐	Retained	Retained in the final 25-item scale after EFA and CFA.
4		Fail to signal before turning left or right.	左转或右转前未发出手势信号。	Removed	Removed after ordinal EFA because the item showed a weak primary loading (0.361). Although it assessed a signalling behaviour, its loading pattern did not support retention within the final four-factor solution.
5		Feel frustrated because of other road users.	因其他道路使用者而感到沮丧。	Removed	Removed after ordinal EFA because, although its primary loading on traffic violations was adequate (0.487), the item primarily assessed frustration or an emotional state rather than an overt aberrant riding behaviour; therefore, its empirical factor assignment was inconsistent with its conceptual content.
6	7	Feel angry at other road users and exhibit aggressive behaviour.	对其他道路使用者感到愤怒并表现出攻击性行为	Retained	Retained in the final 25-item scale after EFA and CFA.
7	22	Nearly lose control due to road obstacles.	因道路障碍物而险些失去控制	Retained	Retained in the final 25-item scale after EFA and CFA.
8	11	Fail to notice the vehicle ahead due to distracted behaviour or inattention.	因分心或注意力不集中而未注意到前方车辆	Retained	Retained in the final 25-item scale after EFA and CFA.
9	4	Ride into restricted areas without permission from traffic signs.	无视交通标志进入禁行区域	Retained	Retained in the final 25-item scale after EFA and CFA.
10	17	Make phone calls while riding.	骑行时打电话	Retained	Retained in the final 25-item scale after EFA and CFA.
11	13	Barely have time to notice someone emerging from behind parked vehicles.	几乎来不及注意到从停放车辆后方出现的人	Retained	Retained in the final 25-item scale after EFA and CFA.
12	1	Speed up to pass through before the traffic light turns red.	在交通信号灯变红前加速通过	Retained	Retained in the final 25-item scale after EFA and CFA.
13	20	Ride with at least one hand off the handlebars.	骑行时至少有一只手离开车把	Retained	Retained in the final 25-item scale after EFA and CFA.
14	9	Suddenly overtake forcefully when riding at the same speed as other vehicles.	与其他车辆同速行驶时突然强行超车	Retained	Retained in the final 25-item scale after EFA and CFA.
15	19	Chat with companions while riding, which distracts attention.	骑行时与同伴交谈，导致注意力分散	Retained	Retained in the final 25-item scale after EFA and CFA.
16	2	Run a red light.	闯红灯	Retained	Retained in the final 25-item scale after EFA and CFA.
17	8	Get angry at other road users and adopt aggressive riding styles.	对其他道路使用者发怒并采取攻击性骑行方式	Retained	Retained in the final 25-item scale after EFA and CFA.
18	5	Fail to use the dedicated bicycle lane (if available).	未使用自行车专用道（如有）	Retained	Retained in the final 25-item scale after EFA and CFA.
19	21	Slip on wet road surfaces or manhole covers.	在湿滑路面或井盖上打滑	Retained	Retained in the final 25-item scale after EFA and CFA.
20		Weave between vehicles.	在车辆之间穿行。	Removed	Removed during expert review because the behaviour was highly dependent on traffic density, road design, available road space, and route conditions and was therefore unlikely to represent a stable individual riding tendency across contexts.

Original No.	Final No.	Original BRBQ item (English)	Chinese version of the BRBQ	Status	Rationale for retention/exclusion
21	12	Fail to notice pedestrians crossing the road when turning.	转弯时未注意到正在过马路的行人	Retained	Retained in the final 25-item scale after EFA and CFA.
22	15	Barely avoid colliding with vehicles that have right of way because of failing to see the yield sign.	因未看到让行标志而险些与拥有优先通行权的车辆相撞	Retained	Retained in the final 25-item scale after EFA and CFA.
23	6	Ride too close to the vehicle ahead, making it difficult to avoid in emergency situations.	骑行时与前车距离过近，紧急情况下难以避让	Retained	Retained in the final 25-item scale after EFA and CFA.
24	3	Ride in the opposite direction.	逆向骑行	Retained	Retained in the final 25-item scale after EFA and CFA.
25		Ride in a lane with fast-moving traffic.	在有快速行驶车辆的车道内骑行。	Removed	Removed during expert review because the behaviour was strongly influenced by road design, bicycle-lane availability, and route choice, resulting in limited contextual representativeness.
26	25	Have difficulty controlling the speed or direction of the bicycle when going downhill.	下坡时难以控制自行车的速度或方向	Retained	Retained in the final 25-item scale after EFA and CFA.
27	14	Fail to notice pedestrians waiting to cross the road at the crosswalk.	未注意到在人行横道处等候过马路的行人	Retained	Retained in the final 25-item scale after EFA and CFA.
28	23	Do not know which gear to use (for variable-speed bicycles).	不知道应使用哪个挡位（针对变速自行车）	Retained	Retained in the final 25-item scale after EFA and CFA.
29	24	Have difficulty maintaining balance at low speeds.	低速骑行时难以保持平衡	Retained	Retained in the final 25-item scale after EFA and CFA.
30	18	Send text messages while riding.	骑行时发送短信	Retained	Retained in the final 25-item scale after EFA and CFA.
31	10	Fail to yield to pedestrians.	未礼让行人	Retained	Retained in the final 25-item scale after EFA and CFA.
32		Attempt to ride with the rear wheel lifted off the ground as a stunt.	尝试以抬起后轮的方式进行特技骑行。	Removed	Removed after ordinal EFA because it showed an ambiguous loading pattern, with a primary loading of 0.399 on distracted behaviours and a secondary loading of 0.327 on traffic violations. The item represented stunt riding rather than distraction, reducing conceptual coherence.
33		Chase another cyclist or road user after becoming angry with them.	生气后追赶另一名骑行者或其他道路使用者。	Removed	Removed during expert review because it overlapped conceptually with retained anger- and aggressive-riding items; its removal reduced content redundancy.
34		Ride under the influence of alcohol or drugs.	饮酒或使用药物后骑行。	Removed	Removed during expert review because it represented a relatively infrequent and sensitive risk behaviour and was potentially susceptible to underreporting because of social-desirability bias.

Note. Items 20, 25, 33, and 34 were removed during expert review. Items 1, 2, 4, 5, and 32 were removed after the ordinal EFA in Sample 1 ($n = 241$) based on loading patterns and conceptual interpretability. Final item numbers refer to the retained 25-item Chinese BRBQ.

Supplementary Table S2. Final 25-item Chinese and English BRBQ with factor assignment and scoring information

Instructions. Please indicate how often you engage in each behaviour while cycling. Responses are recorded on a five-point scale: 1 = never, 2 = rarely, 3 = sometimes, 4 = often, and 5 = nearly all of the time.

指导语。请根据您在骑行过程中从事以下每种行为的频率进行作答。采用五点评分：1 = 从不，2 = 很少，3 = 有时，4 = 经常，5 = 几乎总是。

Scoring. Calculate the mean of the items assigned to each of the four subscales. Higher scores indicate more frequent aberrant cycling behaviour within the corresponding domain. The four subscale scores should be reported separately.

计分。分别计算四个分量表所属条目的平均分。分数越高表示相应维度的异常骑行行为越频繁。四个分量表分数应分别报告。

Final item no.	Factor	因子	English item	中文条目
1	Traffic violations	交通违规	Speed up to pass through before the traffic light turns red	在交通信号灯变红前加速通过
2	Traffic violations	交通违规	Run a red light	闯红灯
3	Traffic violations	交通违规	Ride in the opposite direction	逆向骑行
4	Traffic violations	交通违规	Ride into restricted areas without permission from traffic signs	无视交通标志进入禁行区域
5	Traffic violations	交通违规	Fail to use the dedicated bicycle lane (if available)	未使用自行车专用道（如有）
6	Traffic violations	交通违规	Ride too close to the vehicle ahead, making it difficult to avoid in emergency situations	骑行时与前车距离过近，紧急情况下难以避让
7	Traffic violations	交通违规	Feel angry at other road users and exhibit aggressive behaviour	对其他道路使用者感到愤怒并表现出攻击性行为
8	Traffic violations	交通违规	Get angry at other road users and adopt aggressive riding styles	对其他道路使用者发怒并采取攻击性骑行方式
9	Traffic violations	交通违规	Suddenly overtake forcefully when riding at the same speed as other vehicles	与其他车辆同速行驶时突然强行超车
10	Traffic violations	交通违规	Fail to yield to pedestrians	未礼让行人
11	Notice failures	注意失败	Fail to notice the vehicle ahead due to distracted behaviour or inattention	因分心或注意力不集中而未注意到前方车辆
12	Notice failures	注意失败	Fail to notice pedestrians crossing the road when turning	转弯时未注意到正在过马路的行人
13	Notice failures	注意失败	Barely have time to notice someone emerging from behind parked vehicles	几乎来不及注意到从停放车辆后方出现的人
14	Notice failures	注意失败	Fail to notice pedestrians waiting to cross the road at the crosswalk	未注意到在人行横道处等候过马路的行人
15	Notice failures	注意失败	Barely avoid colliding with vehicles that have right of way because of failing to see the yield sign	因未看到让行标志而险些与拥有优先通行权的车辆相撞
16	Distracted behaviours	分心行为	Listen to music while riding	骑行时听音乐
17	Distracted behaviours	分心行为	Make phone calls while riding	骑行时打电话
18	Distracted behaviours	分心行为	Send text messages while riding	骑行时发送短信
19	Distracted behaviours	分心行为	Chat with companions while riding, which distracts attention	骑行时与同伴交谈，导致注意力分散
20	Distracted behaviours	分心行为	Ride with at least one hand off the handlebars	骑行时至少有一只手离开车把
21	Control errors	操控错误	Slip on wet road surfaces or manhole covers	在湿滑路面或井盖上打滑

Final item no.	Factor	因子	English item	中文条目
22	Control errors	操控错误	Nearly lose control due to road obstacles	因道路障碍物而险些失去控制
23	Control errors	操控错误	Do not know which gear to use (for variable-speed bicycles)	不知道应使用哪个挡位（针对变速自行车）
24	Control errors	操控错误	Have difficulty maintaining balance at low speeds	低速骑行时难以保持平衡
25	Control errors	操控错误	Have difficulty controlling the speed or direction of the bicycle when going downhill	下坡时难以控制自行车的速度或方向

Factor composition. Traffic violations: Items 1–10; Notice failures: Items 11–15; Distracted behaviours: Items 16–20; Control errors: Items 21–25.
因子构成：交通违规：条目1–10；注意失败：条目11–15；分心行为：条目16–20；操控错误：条目21–25。
